# Chronic pain as a blind spot in the diagnosis of a *depressed society*. On the implications of the connection between depression and chronic pain for interpretations of contemporary society

**DOI:** 10.1007/s11019-022-10109-9

**Published:** 2022-08-11

**Authors:** Dominik Koesling, Claudia Bozzaro

**Affiliations:** Department of Medical Ethics at CAU Kiel, Kiel, Germany

**Keywords:** Depression, Chronic pain, Interconnection of depression and chronic pain, Contemporary society

## Abstract

One popular description of current society is that it is a *depressed society* and medical evidence about depression’s prevalence may well make such an estimation plausible. However, such normative-critical assessments surrounding depression have to date usually operated with a one-sided understanding of depression. This understanding widely neglects the various ways depression manifests as well as its comorbidities. This becomes evident at the latest when considering one of depression’s most prominent and well-known comorbidities: chronic pain. Against this background, we aim in this article to substantiate our leading claim that the phenomenal interconnection between depression and chronic pain must be acknowledged in the global diagnosis of a depressive society. Thus, we argue here for a complementation of the dominant interpretation of a depressed society. This would support the overcoming of oversimplified images and estimations about depression in current society and further, help to recognize chronic pain properly on the larger scale of assessments that address society as a whole.

## Introduction: A depressed society – an incomplete assessment of contemporary society

In contemporary parlance, the term *depression* can have a variety of meanings. For example, a depression can be a weather phenomenon, an economic situation or a hollowed part of a surface. Nowadays, though, depression is without doubt best-known and dominantly used for talking about a condition people experience which contains negative-pathological connotations. In this specific meaning the term’s usage ranges from rather subjective articulations like ‘be somewhat depressed’ or ‘feel a bit depressed at the moment’ up to medical diagnoses such as a *depressive episode* or *chronic depression*. In this latter meaning depression and its various aspects have for some years now been a central topic and subject of intense debates in the humanities and social sciences. Here, in light of increasing numbers of people being diagnosed with clinical depression, some authors have examined possible connections between depression and contemporary society. Some have posed the question of whether society actually evokes or increases depression, like Summer did in her 2008 work *Macht die Gesellschaft depressiv?* which translates best as: *Does society make people depressive?*^1^, for example. A consensus has not yet been reached regarding this very question or the more general one about the possible connections between depression and contemporary society. As a result, the debate is still ongoing.

In this specific debate, however, one author’s work in particular stands out. Ehrenberg’s much-discussed *The Weariness of the Self: Diagnosing the History of Depression in the Contemporary Age* (2016) has been fundamental for the whole complex of questions on the connections between depression and contemporary society. First published in 1998 with its original title *La fatigue d’être soi: Dépression et société*, this work has since been republished and translated several times and built upon by other authors like Han (2016). Ehrenberg’s writing laid groundwork for the discussion of whether we nowadays live a depressed society. In it, Ehrenberg (2016: 185) portrayed the increasing necessity of “[s]elf-control, flexibility of mind and feeling, and the capacity for action[, which] meant that each individual had to be up to the task of constantly adapting to a changing world that was losing its stable shape, becoming temporary, consisting of ebb and flow, something like a snakes-and-ladders game.”

Thus, he has contributed to one of the most prominent and efficacious propositions on how to grasp current times. The permanent work of self-improvement, optimization, customization, decision making, and proving one’s self – especially if combined with feelings of insecurity, inadequacy and ‘not being good enough’ – inheres an escalating, overwhelming moment. This is because these demands are interminable and not only address individual subjects in a specific social role they have to oblige, but in their entire personhood (cf. King et al. 2018, esp. 252–255; Bröckling 2013: 283–284). It is crucial to acknowledge that these demands, expectations and imperatives of people like accomplishing more (and more) are not only being imposed upon them. Rather, people themselves also foster and reproduce such demands by internalizing them and thereby, make them their own. This way, these demands become the expectations that individuals have of themselves (cf. Fuchs et al. 2018: 9). Particularly, the said normative demands, expectations and imperatives, which are an inscription of modern societies are difficult or even impossible for an individual to escape.

Exactly this has led the debate to intensify its focus on specific areas of society – especially the world of labor – or society as a whole as being a possible cause of depression. Estimations as such have fueled one leading thesis in the discourse about depression, namely: modern society does not accidentally cause depression. Instead, it occurs systematically. If such an interpretation is deemed plausible, for example, when it can be shown concretely, “that overload at work […] contributes to the increasing prevalence of depressive disorders in the workforce to a relevant extent” (Siegrist 2018: 221)^2^, then depression is by no means merely a disease an individual simply ‘has’ or ‘gets’^3^, but a result of societal developments. It is certainly possible to take a critical stance on such bold assessments about the connection of society and depression and oppose them, as some authors, such as e.g. Ingenkamp (2012) have done. Nevertheless, it is unmistakably a merit of the highlighted normative-critical contributions to the debate that depression is no longer solely discussed as an individual medical condition, but as a socio-structural phenomenon as well.

In spite of this immense achievement, even many of these contributions share the major flaw of understanding depression as an “‘inner’, mental, and individual disorder” (Fuchs 2013: 220), i.e. that they do not only – consciously or not – buy into a body-mind-dualism but also into understanding depression as a disease of the latter essentially, rendering other facets that can accompany it or it may manifest itself in as a mere optional addendum. This is evidenced by the fact that important counterparts of depression like chronic pain are of no concern within the mentioned societal diagnosis. Based on the medical findings and knowledge on the interconnection of depression and chronic pain, we claim that such a lack of properly understanding and integrating chronic pain, which is a well-known comorbidity of depression, is one decisive blind-spot of the diagnosis of a depressive society. Continuing to disregard this perpetuates two major problems, we try to address with our arguments: on the one hand, the illustrated diagnosis of depression remains deficient as it is one-sided and neglects possible somatic dimensions of depression. On the other hand, the phenomenon of chronic pain is interpreted in a reductionist manner – as merely an individual phenomenon and problem – even though it is also a social and even societal one. These two problems in turn foster false understandings of the issues of depression and chronic pain which have consequences for health policy discourses and their respective everyday handling as well. Because these reductionist interpretations not only influence the perception of both conditions, but also impact how the suffering of those affected by depression and chronic pain is addressed and mitigated.

To substantiate these claims, we make our argument in four steps. First, we engage in a short, general discussion about the relevance of so-called *Zeitdiagnosen* in the social sciences and humanities. Taking this meta-perspective makes it necessary in our second step to backtrack to the medical debate on the already addressed phenomenon of depression and the discourse on chronic pain. Here, we draw attention to the enormous prevalence and increasing incidence of both diseases and especially examine the phenomenal interconnection they share. Building on this, we argue in step three that the phenomenal interconnection calls for a correction in the normative-critical discourses about contemporary society. This should aim to overcome not only the predominant, inadequate understanding of depression, but also the shortfall in addressing chronic pain within these discourses at all. Finally, our fourth step presents our concluding argument.

## *Zeitdiagnosen* as a controversial medium to reflect on society

Taking on a meta-perspective, evaluations such as the mentioned depressed society, which are generally called *Zeitdiagnosen* in German, can be understood as a “[n]ormative and empirical analysis and description of societal development tendencies and problem areas as well as pathologies and dysfunctions” (Rosa/Oberthür 2020: 16)^4^. Often, they focus on *one* phenomenon that is identified as a basic characteristic of the respective present times or even perceived as a sign of an age or epoch. In principle, such evaluations can be regarded as a form of self-description, and often as a self-problematization of the respective present time or *Zeitgeist,* and serve as a medium for societal (self) reflection. In their historical genesis such evaluations are “a child of the 19th century during the era of developed industrialism where a way of *culture-critical thinking* began to measure the high expectations of Enlightenment against the backdrop of a dreary reality, characterized by urbanization, factory work, pauperism, and hygienic problems.” (Bogner 2018: 17, italics in original, cf. 13, 18)^5^ Nowadays, such assessments about the state of current society are widespread and can be found, for example, in feature articles, literature, art, and academia. As a result, there usually are several different perspectives on the respective contemporary society and this pluralism is also reflected in the interpretations, particularly in the socio-theoretical and normative-critical discussions. While such assessments about the state of current societies are sometimes complementary, they do typically compete with each other about interpretive authority. However, despite all the plurality and possible competition, all these interpretations about the state of current society do share the same ambition: to make an accurate estimation about a key or essential feature of an entire society. Therefore, these kinds of diagnoses do not focus on a singular part of social life or just some marginal, individual occurrence. Rather, they make an assessment of society in general (cf. Bogner 2018: 12–13, 17; Volkmann 2015: 144)

*Zeitdiagnosen* identify “the characteristic specifics of a *historically determined* social formation” (Rosa 2020: 223, italics in original)^6^. Thus, they can – as the German term indicates – achieve the taking-up of a notion of Hegel (2009 [1821]: 15) for philosophy and grasp their respective time in thought. Acknowledging this fact leads to three relevant characteristics of *Zeitdiagnosen*. Regardless of how the present is concretely understood, *Zeitdiagnosen* (a) are *topical* in that they describe the *present society*. Further, they (b) *condense* the description to a few central aspects with the aim of addressing a core aspect of society – where primarily those phenomena identified as problematic are brought to attention as they negatively irritate an expected normal state. Correspondingly, there is (c) an implicit or explicit normative, mostly pejorative *evaluation* of said phenomena and societies. Furthermore, such assessments can optionally (d) provide an *attempt to explain* how or why the respective social condition or development came to be (cf. Vogelmann 2019: 620–622; Bogner 2018: 12–13, 18–19; Volkmann 2015: 143–145). *Zeitdiagnosen* can thus provide powerful and impactful interpretation patterns that affect not only various aspects of society like policy or medicine. They also influence perceptions of daily life through their analytical or normative interpretations of the present.

However, at the same time, *Zeitdiagnosen* are controversial as they are situated between a well-founded analysis on the one hand and a convincing generalization on the other. Ultimately, they can become deficient “products of the over-generalizing of societal developments […], that only have a limited scope, whether in historical or social terms.” (Honneth 1995: 7; cf. Volkmann 2015: 145, 147, 149; Kneer et al. 2001: 8) While this can, without a doubt, make a *general* aspect for critique for any of those assessments, it is not of specific interest here. As mentioned above, it is a merit of these normative-critical debates to open the perspective about the influence and interaction between society and individual experiences, such as diseases. Hence, our critique on the diagnosis of a depressive society is not about whether society can adequately be described as depressed, but rather what has to be recognized when describing society in such a way. Therefore, as a starting point, we accept the estimation of a depressive society as one plausible interpretation of current times. As a result, the critique we present in the following focuses solely on the concrete content used for the assessment. To make this even more precise: Especially when accepting depression as a core aspect of contemporary society, one has to properly reflect on this phenomenon to integrate it in the normative-critical discussions in humanities and social sciences. To do so, it is helpful to first take a closer look at depression itself as it is discussed in medical contexts.

## Insights from the medical discourses about the depressive society

Empirical indicators supporting the diagnosis of the current society being a society of depression are quite easily identifiable by taking a close look at the global prevalence of depression. Even before the current COVID-19-pandemic, which has, according to a recent estimation by the COVID-19 Mental Disorders Collaborators (2021, esp. 1700, 1705–1707, 1710), led to an increase in this regard, depressive disorders had immensely affected people globally. As James et al. (2018: 1817) reported, more than 264 million people suffer from depression worldwide. Accordingly, *depressive disorders* ranked 13th among the causes of *disability-adjusted life years*^7^ provided by the comprehensive *Global Burden of Disease Study 2019*. According to these findings, depressive disorders have – from 1990 onwards – risen from 8th to 4th place among 10 to 24-year-olds and for the cohort of the 25 to 48-year-olds it has gone up from number 8 to number 6 (cf. Vos et al. 2020: 1210–1211). In light of such great prevalence, increasing incidence and its vehement impact on health, it is not surprising that by now depression as a disease is acknowledged far beyond health care professionals and academic debates regarding health matters. However, as we mentioned from the beginning, beyond such debates there is often a quite vague usage of the term *depression*. In medicine, however, apart from rather minor disputes, for example, regarding the question, where exactly to draw the line between non-pathological mood changes or grieving and a pathological depression, there is a general consensus about it being a pathology. The “core symptoms […] are depressed mood, and loss of interest or pleasure” (Paykel 2008: 281, italics in original).

In particular, the medical understanding of depression can be illustrated quite well through the common, official medical classification systems found in such works as the *Diagnostic and Statistical Manual of Mental Disorders* (DSM) or the *International Statistical Classification of Diseases and Related Health Problems* (ICD)^8^. The latter classifies depression under *affective disorders* and provides further specification. For example, a one-time occurrence is specified as *F32.- Depressive episode.* Diagnostics has a multi-level graduation that ranges from mild to moderate to severe manifestations. The medical codes of the ICD and DSM already name an entire range of possible and different psychological *and* physical symptoms. Yet one can identify “a clear emotional despondency, or sadness (depressive mood), […] a limited possibility of experiencing joy, fun, lust and interests (anhedonia), and […] reduced drive, less activity and becoming more easily exhausted” (Pössel 2019: 676)^9^ as depression’s essential characteristics. Overall, there has been a broad spectrum of symptoms described for depression, beginning with tiredness, reduction or loss of appetite, sleep disorders, and also agitation, the decline in or loss of the ability to experience emotions and decreased self-esteem and confidence. This characterization is reinforced and expanded when a phenomenologically-oriented perspective is taken into account:

“On the physiological level, it manifests itself in disturbances of the sleep-wake-cycle, the daily hormone, temperature, and activity periodicity and also in a loss of drive, appetite and libido. At the same time, the psychophysical inhibition transforms one’s own body into an alienated object that closes itself off from the environment and resists all future-oriented impulses to act upon. […] People who are depressed fail to get up on time, withdraw from social obligations and experience a permanent feeling of being left behind and excluded. The heavy and stiffened body also loses its bodily-affective resonance: patients are no longer able to be touched and affected by other people or emotional situations. They complain of an agonizing loss of feelings where they are no longer capable of feeling anything even for their next of kin.” (Fuchs 2018: 71–72)^10^

Taking all this into account, depression must – overall and without doubt – be understood as a complex syndrome in which several of the named symptoms can occur in different combinations. It is striking, though, that despite the wide discussion and recognition of depression’s somatic symptoms, the disease is still often understood merely as a mental phenomenon – especially in western cultures. In contrast, depression’s material, physical or bodily manifestations are largely marginalized^11^, which is very misleading as already evidenced by the work of Fuchs (2013, esp. 220–222) for example. Accordingly, depression in its entirety must be taken seriously – if one wants to address it properly and not limit the perspective on its mental, emotional, or psychological manifestations. Especially phenomenological or phenomenologically informed research on depression conducted by authors such as the already mentioned Fuchs or Ratcliffe (2015: 75–98) for instance put special emphasis on the body or the bodily dimension in the experience of depression^12^. Such works serve as an important objection to the common, yet highly reductionist and flawed account of depression by referring to first-person-experiences and -descriptions given by depressed people, which often explicitly point towards the body’s role in depression. Acknowledging this calls for taking a closer look on *Somatic symptoms in depression* (Kapfhammer 2006) such as fatigue or forms of bowel syndromes that can link to depression. It is exactly because of this that one must look at the already mentioned phenomenon of chronic pain as well, which want to do now.

Although, for sure, it is important to acknowledge all the various forms of maladies and diseases depression is accompanied by or can express itself in, chronic pain needs to be in focus here specifically. One of the main reasons for this being the combination of both the repeatedly documented, joint occurrence of both diseases in medical diagnosis on the one hand as well as chronic pains’ high prevalence all around the globe^13^on the other hand. A good place to start elaborating on this in more detail is by taking a glimpse at the relationship between pain and depression in general at first. According to Schmahl and Bär (2017: 690) “[p]rimarily depressed patients complain often about their pain symptoms and chronic patients report a high prevalence of depressive disorders.”^14^ The medical specialist terminology even demonstrates this close link very clearly. For example, it uses terms like the *pain-depression dyad* and, respectively, the *depression-pain dyad* (cf. Li 2015; Chopra/Arora 2014; Baier et al. 2003: 2433). Such terminology is especially justified if one takes a closer look at the relationship between depression and chronic pain. This relationship indicates a close connection between both diseases as “up to 90% of patients with chronic pain suffer from depressive moods and around $${\raise0.7ex\hbox{$1$} \!\mathord{\left/{\vphantom {1 3}}\right.\kern-\nulldelimiterspace}\!\lower0.7ex\hbox{$3$}}$$ of these patients meet the criteria for a severe depressive episode” (Taghizadeh/Benrath 2019: 515)^15^. Although the precise interrelation is still being studied, it is known that both diseases share a very close medical relationship. Indeed, quite often, depression and chronic pain go hand in hand and in many cases one cannot decipher whether depression came before the chronic pain – or vice versa – or whether both conditions have the same origin. Regardless of the concrete genesis, the relationship between depression and chronic pain is medically undisputed, especially since both diseases display some of the same characteristics when considering the impact on patients’ lives. These include, for example, that both conditions are often accompanied by social isolation from family, friends and colleagues and reduced social participation. Furthermore, several findings show reciprocal negative effects for patients as exemplified by Bair et al. (2003: 2435, cf. esp. 2434–2435, 2437–2439, 2441–2442). In their review they summarized that “as the severity of pain increases, depressive symptoms and depression diagnoses become more prevalent.^[…]^ Likewise, as depression symptoms increase in severity, pain complaints are reported more often.” Taking this into account, it appears more than appropriate to consider both phenomena closely in a joint perspective.

Against this background, one can now address chronic pain itself more closely. Terminologically *chronic pain* subsumes a variety of different phenomena such as recurring migraines, back pain with regularly radiating pain peaks, tumor-related constant pain, and the nerve-related, whole-body pain generated by fibromyalgia. According to the common, rather pragmatic definitions identifiable in medicine, pain becomes chronic when it is constantly present or recurring over a three-month period or longer (cf. Treede et al. 2019). This demarcation of what constitutes chronic pain has been widely criticized not only because it is arbitrary, but because it obscures the fact that chronic pain can be quite diverse and manifest in various forms. In fact, chronic pain can be differentiated in various ways depending, for example, upon where the pain originates, it being a type of recurring or constant pain or its respective intensity (cf. Kröner-Herwig 2017: 5–6; Thomm 2016: 132–138). Of particular interest here, however, is the high prevalence of chronic pain, which has been indicated decisively by the *Survey of chronic pain in Europe: Prevalence, impact on daily life, and treatment* (Breivik et al. 2012). In their work, the authors not only illuminated the effects chronic pain conditions have on affected people’s lives and the various associated struggles, but could also show how many people are affected by it. Across Europe and Israel “[t]he prevalence of chronic pain ranged from 12% to 30%, highest in Norway, Poland and Italy, and lowest in Spain, Ireland and the UK” (Breivik et al. 2012: 289). Taking Germany as an example, this means, that even according to conservative estimations far more than 10 million people suffer from chronic pain here. If one follows the extrapolation from Häuser et al. (2013: 49) this figure was even over 23 million in 2013.^16^

However, the prevalence of chronic pain knows no regional or national boundaries and is not even limited to the Western Hemisphere. Chronic pain is a global phenomenon. A convincing indicator among others that underpins this estimation is that low back pain and headache disorders – which by no means have to be chronic, yet commonly are – have shown increased prevalence since 1990 according to the mentioned disability-adjusted life years list in the *Global Burden of Disease* (cf. Vos et al. 2020: 1210). Actually, in a general trend, both of these pain conditions rank in the mid- to top-range of the list; headache disorders rank 15th and low back pain even 9th. With such prevalence, lower back pain even outranks depressive disorders, which, as mentioned, is on the list’s 13th place. Recalling the illustrated medical connection between pain and depression, it is by no means surprising to see pain conditions rank that high. But what does indeed come as a surprise is that the normative-critical assessments about current societies seemingly often fail to recognize the immense social and societal impact chronic pain has in their estimations about current societies. What is even more astonishing is that these assessments that place emphasis on depression usually fail to acknowledge chronic pain at all – and this despite the fact that chronic pain and depression can in many cases be understood as medically connected conditions.

## Chronic pain as a blind spot in the diagnosis of a depressive society

Pain treatment has always played a pivotal role in acute medical treatments and care as well as in preventive medical care contexts. Meanwhile the perception of how to grasp pain is shifting as is exemplified by Conrad and Muñoz (2010: 15): “[W]e mean that pain itself is deemed a medical problem, not just a symptom, sign, or byproduct of another diagnosis.” Nowadays pain treatment and management are increasingly being seen as an independent medical issue which has to be addressed regardless of any possibility of identifying a causing illness or injury. In line with this shift of perception of pain being a medical issue in its own regard and the epidemiological findings, chronic pain has already been regarded as one of the central problems of the present. This holds not only true for the multitude of affected patients, but also for their caregivers, the entire health care system and society as a whole (cf. Kieselbach et al. 2016: 351). In this context, one voice from the medical discourse deserves special attention, namely that of Cousins. Some 23 years ago, he added remarkably to the medical discourse surrounding chronic pain by critically commenting on it and claiming:

“It is appropriate now to regard chronic pain as the silent *epidemic*. The dollar costs now exceed the combined cost of the acquired immune deficiency syndrome, cancer, and heart disease. Patients with chronic pain often suffer silently. Relatives and others are silent; they hope it won’t happen to them. *Society is silent*; mostly it is unaware of this enormous human and financial cost. Politicians are silent because the costs are overwhelming. Finally, there is a huge gap between knowledge and practice, and this gap is, in fact, widening as the knowledge increases almost exponentially.” (Cousins 1999: 540, italics by the authors).

These considerations, which take the immense extent of chronic pains’ prevalence seriously, led Cousins directly to a prognosis that is compatible with the thesis proposed here: “Chronic pain will be regarded as the disease of the 21st century.” (Cousins 1999: 540) To date, though, – and this is quite striking – a connection of Cousins’ thesis’ content within the socio-theoretical and critical discourse has remained largely missing. There have been first efforts to reflect on chronic pain in this regard, but in those efforts, pain is usually solely understood as a social problem with regard to its high economic impact on societies. This frames chronic pain as a problem *for* society. What has been widely neglected until now is a possible shift in perspective that asks whether and in what way chronic pain might be a health issue that occurs *through* or even *because* of society. In such a framework chronic pain would not only appear as a social or maybe societal problem, but it would highlight that it is – just like depression – heavily mitigated and can even be caused by society. As is meanwhile known, factors like under- or mistreatment due to a lack of specialized health care institutions like pain clinics, social behavioral expectations or general pressure to perform can amplify or even cause chronic pain (cf. Aster/Sommer 2019; Koesling et al. 2019). When looking at the socio-theoretical and critical discourse however, Han (2020) positively stands out here as he not only recently addressed “today’s epidemic of chronic pain” (Han 2020: 40)^17^ in a more socio-critical fashion, but also implemented the highlighted connection of depression and chronic pain on a socio-diagnostic level.

The fact that such impulses from the humanities and social sciences, especially those with normative-critical character, remain rare seems to be related to the downright assumption made in these fields that chronic pain is the exclusive responsibility of health sciences and professions. Indeed, to date, Liebsch’s (2007: 65)^18^ assessments about the phenomenon of chronic pain still hold true: “Due to the complexity and the drastic experience of chronic pain, its influence is being handed over to medical and psychological experts and thus, the condition is clearly being medicalized.” However, it is precisely this sole medicalization that proves to be deceptive insofar as chronic pain itself cannot be dealt with by medical pain experts alone. Medicine often reaches its limits when helping patients with chronic pain conditions and despite all efforts cannot provide the desired relief. Additionally, this medicalization can lead to a too narrow approach towards chronic pain in theory and in practice, especially with regard to social aspects of chronic pain. Chronic pains’ complex etiologies, which are recognized in the so-called bio-psycho-social model of pain (cf. Arnold et al. 2014: 459) already hint towards a proper reflection of the social. However, the social and especially the societal aspects of pain still remain widely neglected. If social aspects are addressed at all, they are usually handled secondarily and are limited to quantifiable and socio-economic parameters or to close social relationships with family, friends or co-workers. Far too little consideration is being given to social aspects in a broader scope such as the “upheavals, distortions and tensions in the social framework” (Han 2020: 42)^19^. Despite the *social* generally by no means being limited to the micro-social phenomena, current approaches to chronic pain usually only address social aspects centering on the affected individual, e.g. regarding its employment status or family. Yet, this obscures a discussion about chronic pain’s embeddedness in current society, especially regarding questions about a possible societal causation. This might be understandable when coming from a rather medical point of view on chronic pain at first. However, it has to become irritating in light of known socio-structural facets like the correlation between poverty and chronic pain, which, for example, Feierabend et al. (2018) hinted towards to and especially when reconsidering the normative-critical discussions about depression and both diseases, depression and chronic pain, being linked phenomenally in many ways.

Currently, this puts the discussions about chronic pain in a limited and problematic framework that tends to “privatize and psychologize the suffering that society would be *responsible* for.” (Han 2020: 19, italics by the authors, cf. 20)^20^ However, as we discussed in the case of depression, behavioral norms and societal standards such as permanent and increasing requirements to perform and self-optimize can somatize and also be aggravating or contributing factors to chronic pain. In contemporary times, people are involved in deep conflicts with themselves and “[t]he resulting internal *pressures* plunge the person into depression. These also cause chronic pain.” (Han 2020: 40, italics in original)^21^ The problem arising from this is not aspects such as social exclusion and disregarding, that chronic pain can (also) create or intensify, are being left out of consideration. In fact, these are already being addressed in current discussions. The issue is that society as a co-causation or causation of chronic pain remains hidden and thus, the discussion about chronic pain and its social facets remains superficial. And this despite the fact that, for quite a while now, it has been established, that next to biological and psychological factors, social ones, ranging from a micro-social to macro-social level, play a crucial role in pain becoming chronic or chronic pain’s worsening.

Although perhaps not directly recognizable as explicitly social or societal factors, effects of common work-related conditions such as one-sided, repetitive work processes, unhealthy physical postures during the workday, the feeling of having to work when ill or injured, or actually having to do so because of short sick leave or no sick leave at all, can be factors that promote or generate pain and lead to pain becoming chronic. Hence, by no means are such factors of chronic pain restricted or exclusive to status of unemployment, lower levels of formal education or life-style factors like sports activities, smoking or diets. Because next to those it is norms or common patterns of perception and interpretation of (chronic) pain for instance, which, up until now, have often been neglected or even ignored completely, that that can amplify pain or make it become a chronic condition (cf. e.g., Koesling et al. 2021; 2019; Dorner 2018: 2). Presumably this issue at hand becomes most evident in the various stereotypes ranging from general disbelief about the authenticity of pain conditions and the insinuation that the (supposedly) affected might be trying to scam insurance funds, for instance, to more specific prejudices such as the misbelief that only certain groups of people can be affected by a specific form of pain, such as headache disorders (cf. e.g., Dreßke 2016, esp. 334; Thomas 2000: 684). Not only can such forms of ignorance, prejudices, stereotypes, and stigma be hurtful to those in pain, but they can be accompanied by social exclusion, which itself is known to be a potential trigger for pain. All of this proves all the more tragic for those affected by chronic pain because “[r]esearch has shown that higher levels of social support are associated with lower levels of chronic pain” (MacDonald and Leary 2005: 207; cf. e.g., Zhang et al. 2019: 266–267), making social support from others an already recognized way of potential help.

Especially when bearing the latter aspects in mind, one must acknowledge that solely focusing attention towards treating chronic pain medically cannot be a solution, as by doing so “the palliative society *depoliticizes* pain in that it *medicalizes* and *privatizes* it. The *societal dimensionof pain* is thereby suppressed and repressed.” (Han 2020: 21, italics in original)^22^ Taken together with the current high prevalence of chronic pain, it seems appropriate to at least discuss chronic pain not only as an issue of the current times, but especially as one of current society.

## Conclusion: Depression and chronic pain – two sides of the same coin

We began from the common estimation of current society as depressive that stems from humanities and social sciences and then briefly addressed some general issues and benefits of such normative-critical assessments from a meta-perspective. We then went on to relate back to the medical discourses about depression in order to highlight its current deficit in failing to properly acknowledge depression’s various manifestations. One of those is in fact chronic pain, which is widely recognized as one major medical issue of the present. Using this knowledge from the medical discourse and chronic pain’s enormous prevalence, we argued to integrate it into the discussions about current societies. That chronic pain has not already been recognized in the adjourning normative-critical debates in a more extensive way is surprising. This is especially so due to the knowledge from medical discourses, namely that both conditions often present concurrently. In view of depression, there often remains a “split between somatic or external and mental or internal symptoms” (Fuchs 2013: 221) and a one-sided focus on the latter. By often prioritizing or reducing depression to a mere matter of *mental* health an outdated medical understanding of depression is perpetuated. Therefore, grasping the issue by solely addressing depression one-sidedly and dualistically seems to be a mistake of disregarding key factors. Depression’s somatic aspects also require a closer look, and here chronic pain *must* receive proper notice and attention, especially in the socio-critical discussions. Thus, it seems appropriate to venture a discussion of depression and chronic pain as being mutually *connected problems* due to their inherent relationships.

If one does not want to regard it as a mere coincidence that both diseases share similarities phenomenally and in their respective prevalence, and furthermore, are often even comorbidities, then it is not only plausible, but necessary to apply this knowledge when addressing issues of contemporary society on a larger scale. Specifically, one needs to expand the *Zeitdiagnose* of a depressed society by integrating chronic pain into it as depression and chronic pain can be viewed as two sides of the same coin. As mentioned earlier, it is certainly possible to oppose or be skeptical about such diagnostic estimations in general and those surrounding a depressed society in particular, for example due to objecting to normative-critical assumptions at all. However, if one is not fundamentally averse to it and also finds at least some plausibility in ascribing depression to current society as one of its main issues, then one should have a holistic understanding of depression itself. Consequently, those wishing to address depression in such a manner cannot avoid talking about chronic pain (anymore). Otherwise, it will result in a constricted perspective not only on the phenomenon of depression itself, but on the view of individuals’ suffering in contemporary society. To avoid a reductionist view here, it must therefore be recognized that *the depressed society also suffers from chronic pain.* As presented here, this by no means calls for a replacement of one socio-critical narrative, one *Zeitdiagnose*, with another, but rather an *extension* of one of the prominent narratives regarding current society.

However, acknowledging what has been outlined here can only serve as a starting point laying perspective ground for further questioning on the matter(s) at hand. To possibly adjust the ideas presented here and substantiate them even more, there is need for more detailed research on a theoretical as well as empirical level – ideally even combining both – on the interaction of depression and chronic pain, especially when it comes to its social or societal facets. Because next to the more general considerations here, it would be crucial to know about possible differences that might show up when comparing different cultural or regional backgrounds as well as younger and older generations for instance. It might be case that some are prone to a suffering consisting of a combination of depression and chronic pain, which has been more in focus here, while for others it might be more likely to be affected by one or neither of these diseases. Discussing these aspects, also always taking into accout other current, possibly interacting events such as climate change, war, famine and the housing crises for instance, can provide a more accurate, more refined interpretation of our contemporary society that, among other things, is characterized by depression as well as chronic pain.

## Footnotes

1 For references or quotations, which are originally in German, we will provide translations in the main text and the original in footnotes.

2 „dass Überforderung bei der Arbeit […] zur Ausbreitung depressiver Störungen in der Erwerbsbevölkerung in relevantem Umfang beiträgt.“

3 Although we do not want to specifically address this issue in the paper at hand, this specific phrasing of ‘having’ or ‘getting’ a disease refers to the established difference one can draw between *illness* in the sense of the subjective perception of being sick from a first-person-perspective as well as *disease,* which is a label, presumably objective, ascribed from a third-person-perspective – often done so by professionals such as doctors – resulting in acknowledged diagnoses for instance. However, especially considering both phenomena at center of the subsequent argument, that are depression and chronic pain, time and time again the assessments of illness and disease diverge, sometimes to a very high degree, which by all means constitutes a very important topic. Precisely due to this importance however, this topic deserves research specifically addressing this issue and thus, it is inappropriate for us to engage with this issue alongside our main focus of interest here.

4 „[n]ormative und empirische Analyse und Beschreibung gesellschaftlicher Entwicklungstendenzen und Problemlagen sowie potentieller Pathologien oder Dysfunktionen“.

5 „ein Kind des 19. Jahrhunderts, und zwar jener Ära des entwickelten Industrialismus, in der ein *kulturkritisches **Denken* die hochgespannten Erwartungen der Aufklärungszeit an der tristen, durch Urbanisierung, Fabrikarbeit, Pauperismus und Hygieneprobleme geprägten Realität zu messen beginnt.“

6 „die charakteristischen Spezifika einer *historisch bestimmten* sozialen Formation “.

7 This indicator shows how many healthy years of life are lost due to disease or disability. It is comprised of the *years of life lost* on the one hand and the *years lived with disability* on the other (cf. World Health Organization 2013: 4).

8 Some differences and also problems of both classification systems have been pointed out by Paykel (2008). The primary starting and reference points of his work are the ICD-10 (cf. World Health Organization 1992), which is still valid today, and the meanwhile revised DSM-IV (cf. American Psychiatric Association 1994).

9 „eine deutliche emotionale Niedergeschlagenheit bzw. Traurigkeit (depressive Verstimmung), […] eine eingeschränkte Möglichkeit, Freude, Spaß, Lust und Interesse zu erleben (Anhedonie), und […] ein verminderter Antrieb, weniger Aktivität und eine leichtere Erschöpfbarkeit.“

10 „Auf der physiologischen Ebene manifestiert sie sich in Störungen des Schlaf-Wach-Rhythmus, der täglichen Hormon-, Temperatur- und Aktivitätsperiodik ebenso wie in Antriebs-, Appetit- und Libidoverlust. Zugleich verwandelt die psychophysische Hemmung den eigenen Leib in ein entfremdetes Objekt, das sich von der Umwelt abschließt und allen zukunftsgerichteten Handlungsimpulsen Widerstand entgegensetzt. […] Depressive stehen nicht mehr rechtzeitig auf, ziehen sich von sozialen Verpflichtungen zurück und geraten in ein permanentes Ge-fühl des Zurückbleibens und des Ausgeschlossenseins. Der schwere und erstarrte Körper verliert auch seine leiblich-affektive Resonanz: Die Patienten sind nicht mehr in der Lage, von anderen Personen oder emotionalen Situationen berührt und affiziert zu werden. Sie klagen über eine quälende Gefühllosigkeit, in der sie nicht einmal mehr fähig seien, für ihre nächsten Angehörigen etwas zu empfinden.“

11 Paykel (2008: 287) concludes rightfully: „The modern concept of depression, with emphasis on psychological feelings, is particularly Western, and to some extent a 20th-century development. Earlier Western concepts were less psychological. Some other cultures and languages place emphasis on other aspects.”

12 Although not being at center of the arguments put forward here, one has to acknowledge the social dimension of our experiences as well. After all, all these experiences are shaped or mediated by, intertwined with and constituted in a certain way by the social environment as well as society.

13 For sure one must be very cautious to not make misuse of such an argument and fall trap to reductionist framings such as the highly present, economic one Dorner (2018: 1) hints towards to when acknowledging: “Scientific presentations and stakeholder discussions about pain often begin with epidemiological figures and follow the paradigm: the higher the numbers, the higher the burden for the affected people, the higher the costs for society, and the higher priorities should be given to the problem in the health care planning and financing.” In a reductionist way of perceiving it, chronic pain only appears to be of any relevance because of its economic effects and burden it might, for sure, also be, but not because of the issues it can and does pose itself.

14 „[p]rimär depressive Patienten klagen häufig über Schmerzsymptome, während für chronische Schmerzpatienten eine hohe Prävalenz an depressiven Störungen berichtet wurde.“

15 „[b]is zu 90% der Patienten mit chronischen Schmerzen leiden unter depressiver Verstimmung, und ca. $${\raise0.7ex\hbox{$1$} \!\mathord{\left/{\vphantom {1 3}}\right.\kern-\nulldelimiterspace}\!\lower0.7ex\hbox{$3$}}$$ erfüllen die Kriterien zur Diagnose einer schweren depressiven Episode“.

16 This big difference can be attributed among other things to various methods of collecting data and the respectively applied definition criteria of chronic pain.

17 „[d]ie heutige Epidemie der chronischen Schmerzen“.

18 „Aufgrund der Komplexität und Drastik des Erlebens chronischer Schmerzen ist diese Beeinflussung medizinischen und psychologischen Experten überantwortet und ist so gesehen eindeutig medikalisiert.“

19 „Verwerfungen, Verzerrungen und Verspannungen im sozialen Gefüge“.

20 „[d]as Leiden, für das die Gesellschaft verantwortlich wäre, […] [zu] privatisier[en] und psychologisier[en].“

21 „Die inneren *Pressionen*, die dabei entstehen, stürzen es in die Depression. Sie verursachen auch chronische Schmerzen.“

22 „[d]ie Palliativgesellschaft *entpolitisiert *den Schmerz, indem sie ihn *medikalisiert* und *privatisiert*. Unterdrückt und verdrängt wird dadurch die *gesellschaftliche Dimension des Schmerzes*.“
